# NEIGHBORHOOD QUALITY AND WEEKLY PATTERNS OF PHYSICAL ACTIVITY AMONG BLACK AND WHITE ADULTS

**DOI:** 10.1093/geroni/igad104.2009

**Published:** 2023-12-21

**Authors:** Agus Surachman, August Jenkins, Marina Armendariz

**Affiliations:** Dornsife School of Public Health, Drexel University, Philadelphia, Pennsylvania, United States; University of Illinois at Urbana-Champaign, Urbana, Illinois, United States; University of Texas at San Antonio, San Antonio, Texas, United States

## Abstract

Engaging in regular moderate to vigorous physical activity across the week is recommended for maintaining health. This analysis examined whether objective and subjective neighborhood quality was associated with different patterns of weekly physical activity across the week among middle and older Black and white adults. We leveraged data from the National Study of Daily Experiences (NSDE), in which 833 Black and white American adults (Mage = 62.65; n Black = 167) reported their daily moderate to vigorous physical activity across eight consecutive days. The objective and subjective measures of neighborhood quality were the area deprivation index (ADI) and personal belief in the neighborhood, respectively. Hypotheses were tested using the latent class analysis (LCA) framework. Weekly patterns of daily physical activity were classified into four different groups: Active Every Day (group size = 35.75%), Active on Weekdays (26.63%), Active on the Weekend (11.76%), and Less Active (15.85%). Lower ADI rankings, but not personal beliefs about one’s neighborhood, were associated with a higher probability of being in the three more active relative to the Less Active group, an association stronger among white adults. Finally, the three more active groups showed higher self-rated physical health than the Less Active group. Within-race analyses showed that this association was only evident among white adults. Neighborhood deprivation is an important structural context that may strongly limit adults’ engagement in daily physical health.

